# Silacyclic Derivatives of Heteroaromatic Sulfides as Selective Cholesterol Level Lowering and Vasodilating Agents

**DOI:** 10.1155/MBD.2002.307

**Published:** 2002

**Authors:** Edgars Abele, Kira Rubina, Ramona Abele, Olegs Dzenitis, Pavel Arsenyan, Juris Popelis, Maris Veveris, Dainuvite Meirena, Edmunds Lukevics

**Affiliations:** 1 Latvian Institute of Organic Synthesis 21 Aizkraukles street Riga LV-1006 Latvia

## Abstract

Silacyclic derivatives of heteroaromatic sulfides have been prepared by using phase transfer catalytic (PTC) system thiol / silacyclopropyl iodide / solid K_2_CO_3_ / 18-crown-6 / toluene. The target sulfides were isolated in yields up to 70 %. The S-derivatives of N-methylimidazolyl, benzoxazolyl and 1,3,4-triazolyl thiols selectively lowered the low density lipoprotein (LDL) level in mice with the high cholesterol diet in nutrition.


					Metal BasedDrugs Vol. 8, No. 6, 2002
SILACYCLIC DERIVATIVES OF HETEROAROMATIC SULFIDES AS
SELECTIVE CHOLESTEROL LEVEL LOWERING AND VASODILATING
AGENTS
Edgars Abele, Kira Rubina*, Ramona Abele, Olegs Dzenitis, Pavel Arsenyan,
Juris Popelis, Maris Veveris, Dainuvite Meirena, and Edmunds Lukevics
Latvian Institute ofOrganic Synthesis, 21 Aizkraukles street, Riga, LV-1006, Latvia < kira@osi.lv>
ABSTRACT
Silacyclic derivatives of heteroaromatic sulfides have been prepared by using phase transfer catalytic
(PTC) system thiol / silacyclopropyl iodide / solid K2CO3 / 18-crown-6 / toluene. The target sulfides were
isolated in yields up to 70 %. The S-derivatives of N-methylimidazolyl, benzoxazolyl and 1,3,4-triazolyl
thiols selectively lowered the low density lipoprotein (LDL) level in mice with the high cholesterol diet in
nutrition.
INTRODUCTION
Recently the activity of a wide spectrum of heteroaromatic sulfides on the heart and blood circulatory
system was described [1]. For example, quinoline sulfides exhibit antihypertensive [2-4], vasodilating [2-3],
hypotensive [5, 6], euglicemic and hypolipidemic [7], antithrombotic [8], cardiotonic [9], cardiovascular [10-
12] and cholesterol- [13] and blood sugar lowering [14] activities and may be used in the treatment of
diabetes [15-17] and arthritis [18]. Thiazole and benzothiazole sulfides display vasodilating [19], hypotensive
[20] and antihyperglycemic and antihyperlipidemic [21] activities. Imidazole and benzimidazole sulfides
exhibit vasodilating [22], antihypertensive [23], hypoglycemic [24], antithrombotic [25], cardiotonic [26, 27],
cardiovascular [28], cholesterol [29-31] and blood sugar lowering [32], antidiabetic [33, 34] activities,
increase high density lipoprotein cholesterol over lipid fractions [35] and were used in the treatment of
atherosclerosis [36-39] and osteoarthritis [40]. Triazole sulfides were used in the treatment of high blood
pressure and heart failure [41] and display platelet activator and antithrombotic [42] activities. Oxazole
sulfides exhibit an antihypertensive [43] activity. Indole sulfur derivatives were used in the treatment of
atherosclerosis [44], arthritis [45, 46], diabetic complications [47] and congestive hearth failure [48].
In our previous work the cholesterol level lowering and vasodilating activities of silicon and
germanium containing aliphatic derivatives of heteroaromatic sulfides were studied [1]. The 1-
methylimidazole, benzothiazole and 2-quinoline derivatives exhibited the highest level of activity. The
compounds containing dimethyl(l$-triethylgermylethyl)silylmethyl and dimethyl(l$-
triphenylsilylethyl)silylmethyl substituents were the most active in mice with the high cholesterol diet in
nutrition. It was also shown that aliphatic silyl and germyl 1-methylimidazole derivatives possess a
considerable vasodilating activity.
In continuation of our investigations in the field of cholesterol lowering agents the silacyclic S-
derivatives of N-, O- and S-heterocycles have been synthesized under PTC conditions for the purpose to
increase the heterocyclic sulfides lipophility and selectivity of action on the high and low density
lipoproteins.
MATERIALS AND METHODS
CHEMISTRY
tH NMR spectra were recorded on a Varian 200 Mercury instrument (200MHz) using CDCI3 as
solvent and hexamethyldisiloxane (HMDSO) as internal standard. Mass spectra were registered on a GC-MS
HP 6890 (70 eV). GC analysis was performed on a Chrom-5 instrument equipped with a flame-ionization
detector using glass column packed with 5 % OV-101 / Chromosorb W-HP (80-100 mesh) (1.2 m x 3 mm).
1-(3-Iodopropyl)-l-methylsilacyclopentane and 1-(3-iodopropyl)-l-methylsilacyclohexane were obtained by
Grignard reaction [49, 50].
SYNTHESIS OFSILACYCLIC DERIVATIVES OFHETARYL THIOLS. GENERAL PROCEDURE.
Finely powdered dry K2CO3 was added to a mixture of 10 mmol of thiol (1 7), 10 mmol of 1-(3-
iodopropyl)-l-methylsilacyclopentane or 1-(3-iodopropyl)-l-methylsilacyclohexane and 18-crown-6
(lmmol, 264 mg) in 25 ml oftoluene. The mixture was refluxed with stirring to achieve the disappearance of
the substrates, filtered over the thin silica gel layer and concentrated under reduced pressure. The residue was
purified by column chromatography using benzene-hexane or benzene-ethyl acetate as eluent.
PHARMACOLOGY
Cholesterol level lowering and vasodilating activities and the acute toxicity of synthesized compounds
were determined as described in Ref. 1.
307
Kira Rubina et al. Silacyclic Derivatives ofHeteroaromatic Sulfides as Selective
Cholesterol Level Lowering and VasodilatingAgents
RESULTS AND DISCUSSION
CHEMISTRY
A simple method for the preparation of silacyclic derivatives of the N-, O- and S-heterocyclic thiols
was developed. The phase transfer catalytical system solid K2CO3/18-crown-6/toluene was used (Figure 1).
The use of the stronger base (KOH) led to the destruction of the alkylating agents. The aimed substances
were obtained in good chemical yields (up to 70%) in a short time under mild conditions (Table 1).
3 10 (n=l),
SH Me,,
4
ssj
12
Figure l.
The alkylation of sulfides 5 7 simultaneously containing two SH and NH targets of reaction led to
the S-, N-disubstituted derivatives 13 15 (Figure 2).
SH
N/ -'SH
H
Me
n=1,2
K2CO 18-crown-6 PhMe
...c,,,. -N Me..
Me" 15a
15b
Figure 2.
The 1,2,4-triazole thiol isomerised during the alkylation reaction. Two products have been isolated: 1-
[3-(1-methyl- -silacyclopentyl)propyl]-5-{[3-(1-methyl-1-silacyclopentyl)propyl]thio} 1,2,4-triazole (15a)
in 32% and 3-{[3-(1-methyl-1-silacyclopentyl)propyl]thio}-4-[3-(1-methyl-1-silacyclopentyl)propyl]-1,2,4-
triazole (15b) in 34% yield correspondingly.
308
Metal BasedDrugs Vol. 8, No. 6, 2002
Table 1. Synthesis ofsilacycloallo,l derivatives ofheteroaromatic sulfides 8-14, 15a,b
Thiol
'Reaction time, h, [,, Sulfide ,Isolated yield ,,OZo
1 7 8
2 7 9
3 9 10
72
46
70
70
4 21 i2 20
6 14
7 3
26
62
15a 32
15b 34
The purity of the synthesized compounds was determined by HPLC (<1.5% of impurities). All
substances were mobile oils therefore the elemental analysis was not performed.
The structures and spectral characteristics ofsynthesized substances are shown in Tables 2 and 3.
PHARMACOLOGY
CHOLESTEROL LEVEL LOWERING ,4CTIVITY
The Table 4 data show the serum lipid level at the end of the experiment. The high cholesterol in
nutrition Cholesterol group showed the marked increase in the total and LDL cholesterol in comparison to
the intact control group. The HDL level in Cholesterol group did not differ from the Intact control group.
It has been found, that 2-{[3-(1-methyl-l-silacyclopentyl)propyl]thio}benzoxazole (9), 3-{[3-(1-
methyl-1-silacyclopentyl)propyl]thio}-4-[3-(1-methyl-1-silacyclopentyl)propyl]-1,2,4-triazole (15b), 1-
methyl-2-{[3-(1-methyl-1-silacyclopentyl)propyl]thio}imidazole (8), and 2-{[3-(1-methyl-1-silacyclopentyl)-
propyl]thio}benzothiazole (10) produced a high antiatherosclerotic activity protected against increase in
serum LDL cholesterol level and atherosclerotic coefficient.
Benzoxazole and triazole derivatives 9 and 15b show the highest activity among all the investigated
aliphatic [1] and silacyclic S-substituted heterocycles. A preliminary analysis of the structure-activity
relationship for the cholesterol lowering action clearly indicates the strong influence of the silacyclic
substituent position in the triazole ring. Compound 15a bearing a silacyclopentyl)propyl group in position
has slight effect on the cholesterol level (K 0.799). On contrary 4-N-substitution leads to a considerable
increasing of activity: 3-{[3-(-methy--siacycpenty)prpy]thi}-4-[3-(-methy--siacycpenty)-
propyl]-l,2,4-triazole (15b) is 9 times more active (K 0.091) than the isomer 15a.
The silacyclic imidazole derivative 8 has better K value (K 0.111) than the corresponding
trimethylsilylpropyl analogue (K--0.453) [1]. Moreover, compound 8 show the tendency to increase the HDL
level. This fact indicates that this compound can possess an additional positive influence.
The activity of the silacyclic substituted benzothiazole 10 (K 0.200) slightly differs from 2-(3-
trimethylsilylpropyl)thiobenzothiazole (K 0.285) [1]. The insertion of a methylene group into the five-
membered silacyclopentane ring leads to decrease of activity (atherogenecity coefficient K=0.200 for 10 and
K=0.401 for 11).
It is significant to note that benzoxazole derivative 9 is 3 times more active than benzothiazole
derivative 10.
V,4SODIL,4TING ,4CTIVITY
The vasodilating activity of the studied compounds in experiments in vivo is presented in the Table 4.
In general the studied silacyclic compounds have a weak influence on vasodilatation even in a 50tag/mL
dose. It was found that imidazole derivative 8 exhibits the highest relaxation effect (18% in 10 lag/mL). It is
more active than aliphatic silicon-containing analogues (12-13%), but less active than dimethyl(l-
triethylgermylethyl)silylmethyl substituted imidazole (22%) ].
309
Kira Rubina et al. Silacyclic Derivatives ofHeteroaromatic Sulfides as Selective
Cholesterol Level Lowering and VasodilatingAgents
Table 2. IH and 3C NMR data ofheteroaromatic sulfides
Cpd
1H NMR, d (ppm, CDCI3 / HMDSO)
8 0.06 (s, 3H, SiCH3), 0.51 and 0.72 (each m, 6H,
10
11
SiCH2), 1.51 and 1.67 (each m, 6H, CH2), 3.03
(t, 2H, J 7.0 Hz, SCH2), 3.60 (s, 3H, NCH3),
7.91-8.05 (m, 2H, imidazole cycle)
0.08 (s, 3H, SiCH3), 0.53 and 0.78 (each m, 6H,
SiCH2), 1.54 and 1.85 (each m, 6H, CH2), 3.31
(t, 2H, J 7.2 Hz, SCH2), 7.2-7.6 (m, 4H,
benzoxazole cycle)
0.08 (s, 3H, SiCH3), 0.53 and 0.78 (each m, 6H,
SiCH2), 1.54 and 1.85 (each m, 6H, CH2), 3.34
(t, 2H, J 8.0 Hz, SCH2), 7.33 and 7.79 (m, 4H,
benzothiazole cycle)
0.005 (s, 3H, SiCH3, 0.65 (m,6H, SiCH2),i.64
(m, 8H, CH2), 3.35 (t, 2H, J 7.4 Hz, SCH2),
7.25, 7.74 and 7.87 (m, 4H, benzothiazole cycle)
-12 0.13 (s, 3H, SiCH3), 0.71 (m, 6H, SiCH2), 1.71
(m, 6H, CH2), 3.07 (t, 2H, J 7.0 Hz, SCH2),
7.40, 8.05 and 8.92 (m, 6H, quinoline cycle)
13 -0.01 and 0.00 (both s, 6H, SiCH3), 0.57 (m,
12H, SiCH2), 1.2-1.8 (each m, 16H, CH2), 3.50
(t, 2H, J 7.0 Hz, SCH2), 4.06 (t, 2H, J 7.2 Hz,
NCH2), 7.24 and 7.74 (m, 5H, indole cycle)
li 0.08 and 0.08 (both s, 6H, SiCH3), 0.51-0.83 (m,
15a
12H, SiCH2), 1.54 and 1.82 (each m, 12H, CH2),
3.42 (t, 2H, J 7.2 Hz, SCH2), 4.07 (t, 2H, J
7.4 Hz, NCH2), 7.22 and 7.68 (m, 4H,
benzimidazole cycle)
0.06 and 0.07(each s, 3H, SiCH3), 0.52 and 0.72
(each m, 6H, SiCH2), 1.53 and 1.78 (each m, 6H,
CH2), 3.22 (t, 2H, J 7.4 Hz, SCH2), 4.02 (t, 2H,
J 8.0 Hz, NCH2), 7.84 (s, H, triazole cycle)
13C NMR, d (ppm, CDCI: /HMDSO)
Het
33.06 (N-CH3),
121.83 (C-5), 129.06
(C-4),141.85 (C-2)
109.73(C-6), 118.28
(C-5), 123.66 (C-7),
124.14 (C-4),141.98
(C-7a), 151.74 (C-3a),
165.17 (C-2)
120.86 (C-6), 121.42
(C-5), 124.04 (C-7),
125.94 (C-4), 135.15
(C-7a), 153.35 (C-3a),
167.28 (C-2)
120.86 (C-6), 121.42
(C-5), 124.04 (C-7),
125.94 (C-4), 135.15
(C-7a), 153.35 (C-3a),
167.28 (C-2)
121.45 (C-5), 123.56
(C-6), 123.75 (C-4),
126.44 (C-3), 128.15
(C-4a), 136.23 (C-7),
138.86 (C-8), 145.45
(C-Sa), 149.04 (C-2)
109.68 (C-5), 119.59
(C-4), 119.76 (C-6),
120.52 (C-3), 121.97
(C-7), 130.13 (C-3a),
132.92 (C-7a), 136.53
(C-2)
108.59 (C-5), 118.13
(C-6), 121.48 (C-7),
121.51 (C-4), 136.12
(C-7a), 143.56 (C-3a),
152.07 (C-2)
143.61 (C-5), 160.94
(C-2)
SR
-3.00, 11.51,
14.43,24.18,
27.16, 37.62
-3.31, 11.60,
14.64, 24.61,
27.25, 35.54
-3.29, 11.64,
14.78,24.58,
27.26, 36.90
-4.97, 11.42,
12.73, 12.78,
24.19, 24.44,
30.10
-3.35, 11.55,
15.02, 23.43,
27.19, 34.38
-4.97, -4.81,
11.42, 12.58,
12.73, 12.78,
13.12,24.19,
24.37, 24.44,
24.73, 30.01,
30.10, 40.20
-3.33, -3.30,
11.52, 11.61,
12.26, 14.70,
24.35, 24.61,
27.23, 27.25,
35.86, 46.95
-3.42, -3.31,
11.49, 11.61,
11.95, 14.58,
24.89, 24.97,
27.21, 27.24,
29.64, 35.52,
52.79
310
Metal BasedDrus Vol. 8, No. 6, 2002
15b 0.05 and 0.07(each s, 3H, SiCH3), 0.52 and 0.73
(each m, 6H, SiCH2), 1.53 and 1.78 (each m, 6H,
CH2), 3.11 (t, 2H, J 7.4 Hz, SCH2), 4.05 (t, 2H,
J 8.0 Hz, NCH2), 7.96 (s, 1H, triazole cycle)
143.61 (C-5), 160.93
(C-2)
-3.41, -3.29,
11.51, 11.62,
11.97, 14.60,
24.89, 24.98,
27.22, 27.25,
35.52, 52.79
Sulfide
10
11
12
13
14
15a
15b
Table 3. Mass spectra ofheteroaromatic sulfides
m/z (relative intensity, %)
254 (M*, 5), 225 (40), 207 (14), 197 (29), 183 (35), 170 (34), 157 (51), i41 (31), 114 (100), 99
(23),.83 (15),.71 (31), 59.(19) 43 (34)
291 (M*, 8), 276 (18), 262 (62), 249 (49),' 234 (62),'220' (100), 207 (85), 194 (48), 178 (40), 167
(13), 146 (69), 133 (57, 122 (64), 99 (89), 91 (18), 83 (15), 71 (100), 59 (50), 43 (.83)
307 (M*, 11) 292 (M -Me, 10), 280 (13), 274 (17), 265 (14), 264 (12), 263 (18), 251 (24), 250
(39), 237 (67), 236 (51), 232 (15), 223 (18), 22 (74), 209 (29), 193 (27), 179 (12), 167 (88), 162
(100), 150 (12), 149 (62), 135 (21), 122 (14), 117 (21), 109 (12), 108 (33), 102 (17), 99 (58), 97
(52), 95 .(13), 75 (28), 69 (26), 59 (44), 45 (76), 43 (72)
321 (M*, 10), 278 (100), 250 (26), 237 (30), 236 (28), 223 (21), 194 (10), 167 (40), 136 (15), 113
(15), 108 (15), 85 (46), 69 (11), 59 (24), 45 (28), 43 (27)
301 (M*, 14), 286 (14), 272 (42), 254 (51), 244 (61), 230 (27), 217 (75), 202 (81), 188 (100), 174
(65), 161 (96), 142 (40), 129 (56), 116 (24), 99 (38), 89 (20), 71 (36), 59 (24), 43 (49)
331 (M*-SiMe(CH2)5, 10), 303 (11), 149 (45), 117 (100), 113 (90), 187 (100), 85 (82), 59 (70)
416 (M*-Me, 5), 415 (14), 304 (8), 261 (31), 248 (37), 234 (13), 191 (9), 150 (25), 113 (38), 85
(1 00), 59 (34), 43 (22)
381 (M*, 21), 380 (M*-I, 6), 351 (18), 340 (18), 324 (10), 310 (12), 298 (18), 284 (15), 282 (14),
264 (12), 242 (42), 212 (17), 210 (14), 200 (35), 186 (17),185 (19), 184 (17), 158 (10), 121 (15),
117 (26), 99 (100), 98 (28), 97.(58),, .85 (15), 71 (78), 59 (35),. 45 (32), 43 (33)
381 (M*, 10), 366 (M*-Me, 10), 353 (20), 325 (60), 344 (26), 339 (16), 325 (18), 324 (32), 320
(14), 305 (16), 292 (18), 282 (16), 268 (12), 242 (15), 240 (15), 226 (16), 212 (27), 200 (15), 186
(16), 170 (15), 142 (15), 128 (10), 117 (34), 99 (100), 97 (64), 85 (20), 71 (85), 59 (32), 45 (27),
43 (3)
Table 4. Lipoprotein level lowering properties, vasodilating activity and acute toxicity ofsilacyclic
derivatives ofheteroaromatic sulfides
N
Cholesterol
8
10
ll
12
13
14
15a
15b
The total cholesterol,
high and low density iipoprotein level
and the atherogenicity coefficient (K)
Total cholesterol HDL LDL K
mg/dl mg/dl mg/dl
124,7 70,7 54,0 0,825
99,9 90,5 9,5 0,111
75,3 70,2 5,1 0,073
99,7 82,9 16,7 0,200
87,1 61,8 25,3 0,401
80,3 49,2 3l, 0,726
89,8 77,5 12,3 0,197
88,7 55,2 33,5 0,643
104,9 59,7 45,3 0,799
91,5 83,7 7,9 0,091
Vasodilating activity
l, mM
10
10
5O
10
5O
10
5O
10
50
10
5O
10
5O
10
50
l0
Relaxation
tt.tt
contraction),
%
18
30*
12
19
6
15
-8
19
-5
12
8
17
3
15
9
20
Acute toxicity
375
>800
> 800
> 800
580
> 600
> 800
> 800
5 760
311
Kira Rubina et al.
Intact
control 69,0 64,9
Silacyclic Derivatives ofHeteroaromatic Sulfides as Selective
Cholesterol Level Lowering and VasodilatingAgents
50 15
4,1 0,065
Control/
3
Solvent
ACUTE TOXICITY
The studied compounds have basically a low acute toxicity (Table 4). Only imidazole sulfide
exhibits a medium level oftoxicity (375 mg/kg).
CONCLUSIONS
A PTC method of synthesis of silacyclic derivatives of the N-, O- and S-heterocyclic sulfides was
elaborated. Nine compounds were synthesized and isolated in the yields up to 70%.
They were studied as serum cholesterol level lowering agents. It has been found, that
silacyclopentylpropylthio-imidazole ($), -benzoxazole (9), -benzothiazole (10) and -1,2,4-triazole (15b)
exhibited a high antiatherosclerotic activity. It protected against increase in serum LDL cholesterol level. A
preliminary analysis of the structure-activity relationship for the cholesterol lowering action clearly indicates
the strong influence of the silacyclic substituent position in the triazole ring. Imidazole derivative $ has
shown the tendency to increase the HDL level. This fact indicates that compound can possess an additional
positive influence. The synthesized sulfides are low toxic compounds with weak vasodilating activity.
ACKNOWLEDGEMENT
The authors thank Latvian Council ofScience for the financial support (Grant N 166).
REFERENCES
1. K. Rubina, E. Abele, P. Arsenyan, R. Abele, M. Veveris, E. Lukevics, Metal Based Drugs, 8, 85 (2001).
2. A. Birch, R. Davies, L. Maclean, K. Robinson, J. Chem. Soc., Perkin Trans. 1, 387 (1994).
3. H. Hidaka, Jap. Patent, 04,330,057 (1992); Chem. Abstr., 119, 139132p (1993).
4. R.V. Davies, K. Robinson, PCT Int. Appl. WO Patent 9,102,724 (1991); Chem. Abstr., 115, 92095d
(1991).
5. Tanabe Seiyaku Co., Ltd., Jap. Patent 8,130,991 (1981); Chem. Abstr., 95, 150717q (1981).
6. Tanabe Seiyaku Co., Ltd., Jap. Patent 57,134,469 (1982); Chem. Abstr., 98, 53714r (1983).
7. B.B. Lohray, V. Bhushan, A.S. Reddy, P.B. Rao, N.J. Reddy, P. Harikishore, N. Haritha, R.K.
Vikramadityan, R. Chakrabarti, R. Rajagopalan, K. Katneni, J. Meal Chem., 42, 2569 (1999).
8. S. Raddatz, K.H. Mohrs, M. Matzke, R. Fruchtmann, A. Hatzelmann, R. Mueller-Peddinghaus, Ger.
Patent 4,139,751 (1993); Chem. Abstr., 119, 271168j (1993).
9. N. Beier, J. Harting, R. Jonas, M. Klockow, I. Lues, G. Haeusler, J. Cardiovasc. Pharmacol., 18, 17
(1991).
10. R. Jonas, I. Lues, N. Beier, M. Klochow, K.O. Minck, Ger. Patent 4,041,074 (1992); Chem. Abstr.,
117,151022u (1992).
11. R. Jonas, I. Lues, N. Beier, K.O. Minck, Eur. Patent 721,950 (1996); Chem. Abstr., 125, 168029q
(1996).
12. M. Kidwai, N. Negi, P. Misra, J. Indian Chem. Soc., 77, 46 (2000).
13. M.P. Deninno, R.B. Ruggeri, R.T. Wester, Jap. Patent 2000 95,764 (2000); Chem. Abstr., 132, 251083t
(2000).
14. Y. Nomura, S. Sakuma, S. Masui, PCT Int. Appl. WO Patent 96 12,719 (1996); Chem. Abstr., 125,
114599g (1996).
15. M.S. Malamas, US Patent 5,037,831 (1991); Chem. Abstr., 115, 280004x (1991).
16. T. Aotsuka, T. Nishio, H. Hosono, Y. Nakamura, T. Matsui, H. Ishikawa, PCT Int. Appl. WO Patent 94
15,934 (1994); Chem. Abstr., 121, 280632z (1994).
17. H. Ishiguro, S. Shimada, M. Setani, Y. Yagi, N. Okane, Y. Saito, N. Takitsu, Jap. Patent 2000 256,327
(2000); Chem. Abstr., 133, 222732u (2000).
18. T. Sohda, M. Makino, A. Baba, Eur. Patent 567,107 (1993); Chem. Abstr., 120, 217712q (1994).
19. M. Kato, S. Nishino, M. Hamano, H. Takasugi, PCT Int. Appl. WO Patent 93 10,097 (1993); Chem.
Abstr., 120, 106772q (1994).
20. S.S.Pharmaceutical Co., Ltd., Jap. Patent 8,209,787 (1982); Chem. Abstr., 96, 217861y (1982).
21. R.M. Hindley, US Patent 5,002,953 (1991); Chem. Abstr., 115, 49719y (1991).
22. J. Frazee, C. Kaiser, L.I. Kruse, Eur. Patent 125,033 (1984); Chem. Abstr., 102, 132034y (1985).
23. S.S.Pharmaceutical Co., Ltd., Jap. Patent 6,075,487 (1985); Chem. Abstr., 103, 142015c (1985).
24. T. Fujita, K. Wada, M. Oguchi, H. Honma, T. Fujiwara, PCT Int. Appl. WO Patent 00 61,582 (2000);
Chem. Abstr., 133, 296438z (2000).
312
Metal BasedDrugs Vol. 8, No. 6, 2002
25. H. Sato, A. Nagai, K. Takayanagi, A. Tatsui, H. Yamada, K. Kojo, N. Narita, Jap. Patent 2001 19,678
(2001); Chem. Abstr., 134, 115957v (2001).
26. A.B. Brukshtus, V.N. Garaliene, A.R. Sirvidite, V.K. Daukshas, Khim.-Farm. Zh., 26, 50 (1992).
27. A.B. Brukshtus, V.No Garaliene, A.R. Sirvidite, V.K. Daukshas, Khim.-Farm. Zh., 29, 30 (1995).
28. F. Kaiwa, M. Shoji, H. Domoto, H. Uchida, R. Yoshimoto, Jap. Patent 05,255,308 (1993); Chem. Abstr.,
120, 245090d (1994).
29. T.P. Maduskuie, Jr., US Patent 5,179,117 (1993); Chem. Abstr., 118, 254933s (1993).
30. N.H. Dyson, J.O, Gardner, A. Prince, D.J. Kertesz, US Patent 5,208,331 (1993); Chem. Abstr., 119,
20341 lh (1993).
31. J.T. Billheimer, P.J. Gillies, C.A. Higley, T.P. Maduskuie, Jr., R.R. Wexler, PCT Int. Appl. WO Patent
91 18,885 (1991); Chem. Abstr., 116, 151761p (1992).
32. H. Iwabuchi, T. Fujiwara, T. Fujita, Jap. Patent 2001 97,954 (2001); Chem. Abstr., 134, 280842m
(2001).
33. H. Yamaga, H. Yamaguchi, K. Maruta, M. Yasuhi, F. Hirota, J. Ihihara, Jap. Patent 2000 109,467
(2000); Chem. Abstr., 132, 279218q (2000).
34. T. Fujita, K. Wada, M. Oguhi, H. Honma, T. Fujiwara, H. IwabuChi, PCT Int. Appl. WO Patent 00
59,889 (2000); Chem. Abstr., 133, 251783j (2000).
35. H. Elokdah, T. Sulkowski, D. Cochran, M.-L. McKan, E. Quint, Bioorg. Med. Chem. Lett., 10, 1791
(2000).
36. J.T. Billheimer, P.J. Gillies, R.R. Wexler, C.A. Higley, T.P. Maduskuie, Jr., Eur. Patent 372445 (1990);
Chem. Abstr., 114, 6503k (1991).
37. R.G. Wilde, PCT Int. Appl. WO Patent 93 23,380 (1993); Chem. Abstr., 120, 217678h (1994).
38. R.G. Wilde, G. Richard, C.A. Higley, J.T. Billheimer, R.R. Wexler, PCT Int. Appl. WO Patent 93
23,381 (1993); Chem. Abstr. 120, 217676f(1994).
39. K. Aoki, K. Aikawa, M. Kawakami, Y. Yan, PCT Int. Appl. WO Patent 01 04,110 (2001); Chem. Abstr.,
134, 115956u (2001).
40. G. De Nanteuil, B. Portevin, A. Fradin, J. Bonnet, Actual. Chim., 48 (2000).
41. W.T. Ashton, M. Maccoss, L.L. Chang, P.K. Chakravarty, C.L. Cantone, W.J. Greenlee, A.A. Patchett,
T.F. Walsh, Eur. Patent 409,332 (1991); Chem. Abstr., 11, 29337u (1991).
42. B.J. Teobald, PCT Int. Appl. WO Patent 01 19,826 (2001); Chem. Abstr., 134, 237500m (2001).
43. E.R. Larson, US Patent 5,175,179 (1992); Chem. Abstr., 115, 139845c (1993).
44. R. Steffan, A.A. Failli, PCT Int. Appl. WO Patent 99 11,618 (1999); Chem. Abstr., 130, 209600t (1999).
45. K. Luk, P.E. Mahaney, S.G. Mischke, PCT Int. Appl. WO Patent 00 35,906 (2000); Chem. Abstr., 133,
58710n (2000).
46. N.J. Bach, S.E. Draheim, R.D. Dillard, E.D. Mihelich, J.S. Sawyer, D.W. Beight, M.L. Phillips, T.
Suarez, D.J. Sail, J.A. Bastian,, M.L. Denney, G.A. Hite, M.D. Kinnick, R.T. Vasileff,, M.J. Morin, Jr.,
H.-S. Lin, M.E. Richett, R.W. Harper, J.M. McGill III, B.A. Anderson, N.K. Ham,, R.J. Loncharich,
R.W. Schevitz, US Patent 6,177,440 (2001); Chem. Abstr., 134, 131518h (2001).
47. M.L. Jones, D. Gunn, J.H. Jones, M.C. Van Zandt, PCT Int. Appl. WO Patent 99 50,268 (1999); Chem.
Abstr., 131, 257442k (1999).
48. F. Watanabe, T. Takefumi, H. Tsuzuki, T. Shimamura, PCT Int. Appl. WO Patent 00 15,213 (2000);
Chem. Abstr., 132, 236806n (2000).
49. J.W. Wilt, C.F. Dockus, J. Amer. Chem. Soc., 92, 5813 (1970).
50. N.S. Nametkin, K.S. Vdovin, K.S. Pushchevaya, V.I. Zavyalov, Izv. Akad. Nauk SSSR, Otd. Khim. Nauk,
1453 (1965).
Received- January 9, 2002- Accepted" January 15, 2002-
Accepted in publishable format- April 15, 2002
313

				

